# A longitudinal investigation of gut microbiota dynamics in laying hens from birth to egg-laying stages

**DOI:** 10.5713/ab.24.0889

**Published:** 2025-04-11

**Authors:** Seojin Choi, Eun Bae Kim

**Affiliations:** 1 Department of Applied Animal Science, College of Animal Life Sciences, Kangwon National University, Chuncheon, Korea; 2 Institute of Animal Life Science, Kangwon National University, Chuncheon, Korea

**Keywords:** 16S rRNA Gene, Gut Microbiota, Laying Hens, Longitudinal Study, Machine Learning

## Abstract

**Objective:**

Laying hens are a critical resource in global agriculture, valued for their egg production, which provides an economical and nutritious source of protein. This study aims to comprehensively characterize the developmental changes in the gut microbiota of Hy-Line Brown laying hens from birth to post-laying stages.

**Methods:**

A total of 100 Hy-Line Brown laying hens were reared under controlled conditions, and feces and ileal contents were collected at three post-laying stages (151, 302, and 422 days). DNA was extracted from the samples and the V4 region of the 16S rRNA gene was sequenced using an Illumina MiSeq platform. The Random Forest algorithm was applied to identify microbial predictors and explore their relationships with age.

**Results:**

The rapid increase in body weight continued until day 151, after which it stabilized through day 422. Fecal microbiome diversity increased until day 302, whereas in the ileal content, it grew until day 101 before declining. Throughout all stages, Firmicutes, Proteobacteria, and Bacteroidetes were the predominant phyla. *Lactobacillus* abundance peaked on day 10 (33.75%) across all sampling sites, whereas *Escherichia-Shigella* reached its maximum on day 21 but gradually diminished to 2.88% by day 422 (p<0.05). Machine learning analysis revealed that *Candidatus* Arthromitus and *Clostridia vadinBB60* group consistently had the highest importance scores across all sample sites. At 302 day, body weight exhibited negative relationships with feces *Brevibacterium* (R = −0.87; p<0.05) and *Brachybacterium* (R = −0.79; p<0.05).

**Conclusion:**

This study examined gut microbiota changes in Hy-Line Brown hens from birth to one-year post-laying, highlighting the impact of calcium-rich layer diets. The findings provide insights into microbiota dynamics and their relationship with age, which can be applied to optimize dietary strategies and improve laying hen productivity and health.

## INTRODUCTION

Laying hens play a significant role and represent a substantial proportion of the global agricultural sector. The birds are greatly appreciated for their ability to lay eggs, which offer a nutritious and affordable source of animal protein to people worldwide. Eggs serve as crucial components in numerous culinary traditions worldwide and offer a wealth of essential nutrients. Egg production by laying hens is an economically significant resource. Hens typically commence egg-laying around four months after hatching and can sustain production throughout the year. Owing to laying hens having a longer lifespan than broilers, effective health management is essential [1].

Recent studies have expanded our knowledge of the microbiota (a diverse ecosystem of microorganisms, including bacteria, fungi, and viruses) found in specific environments. This expanding area of study extends beyond the well-researched human microbiome to encompass microbial communities present in farm animals. A crucial subset of these populations, the gut microbiota, located within the digestive system, serves an essential function in the breakdown and uptake of nutrients, thereby influencing the overall well-being and efficiency of livestock. The intestinal microbial ecosystem significantly affects animal health and productivity [2].

Machine learning (ML) has become an invaluable tool for addressing the complexities of microbial communities, employing advanced algorithms to reveal hidden patterns and predictive relationships in vast datasets. Using ML techniques, researchers have gained a deeper understanding of the intricate relationships between gut microbiota and host physiology [3].

This study focused on Hy-Line Brown laying hens, recognized globally as the most balanced brown egg layer [4]. As with other livestock, the importance of the gut microbiota in laying hens is increasingly acknowledged within the scientific community [5]. Research has shown that gut microbiota influence hen health and productivity, including their immunity, growth, and egg quality. A well-balanced microbiota can mitigate the risk of foodborne diseases, thus playing a valuable role in food safety. Moreover, a comprehensive understanding of how the gut microbiota evolves over time is crucial for optimizing interventions to enhance poultry health. These include the strategic use of feed additives, such as probiotics and prebiotics, at various growth stages to promote healthy egg production [6]. The novelty of this study lies in its longitudinal analysis of gut microbiota from hatching to one year post-laying, providing insights into dietary transitions and growth stages on microbiota composition. Research focusing on the basic physiological processes of livestock, particularly analyzing the intestinal microbiota of laying hens across different stages of growth, has been scarce, despite their importance. To address this gap, our study proposes that the gut microbiota of laying hens undergo dynamic changes that are closely linked to their overall health and development.

Analysis of gut microbiota from hatching to before laying [7] revealed that the pre-oviposition gut microbiota varied with feed and that differences in growth were also affected. However, at the beginning of laying, the composition of the layer feed undergoes a critical change, and eggs are produced. Therefore, the analysis was extended to include the post-laying period. Consequently, this study conducted a detailed monitoring of the gut microbiota at seven critical stages, from birth to laying, with the aim of observing and characterizing developmental changes in the microbiome of poultry.

## MATERIALS AND METHODS

### Animal trial

A total of 100 Hy-Line Brown laying hens were acquired from Korea Poultry Co., located in Anseong, Korea. The birds were reared on a farm in Chuncheon, Korea, in compliance with Korean animal welfare guidelines. The hens were provided with a commercial feed formulated to meet the nutritional requirements of their specific growth phases. The layer diets were provided *ad libitum* (Table 1) with free access to water. The pen environment, including the lighting, water supply, ventilation, and temperature control, was managed using a commercial smart switch. The temperature and humidity were continuously monitored (ESP-01), and real-time data were collected using a web server (Supplement 1). Eggs were collected an average of three times per week to measure the daily laying rate (Supplement 2). All experiments were approved by the Institutional Animal Care and Use Committee (IACUC) of Kangwon National University (KW-210802-3).

### Sample collection

In our previous study, the sampling was conducted at four growth stages (10, 21, 58, and 101 days), focusing on the pre-laying period of Hy-Line Brown laying hens. Building on this foundation, the current study extends the analysis to include these three additional sampling points during the post-laying period (151, 302, and 422 days), resulting in a total of seven growth stages being analyzed. Sampling was conducted in three stages based on the age after laying, specifically at 151, 302, and 422 d. Birds were selected to be representative of the body weight of the entire population at each sampling point. For feces collection, birds were isolated in a clean plastic pen floored with sterilized aluminum foil, and weighed. Nine birds were selected to represent the overall body weight distribution of the chickens and were euthanized using CO_2_ asphyxiation. The ileal contents were collected on each sampling day. Briefly, Using a sterile tip, 1 g of feces matter and ileal contents were transferred into 1.7 mL tubes. A total of 53 feces samples and 27 ileal contents samples were preserved at −70°C pending DNA extraction (Supplement 3).

### DNA extraction and 16S rRNA sequencing

Fecal and ileal samples (250 mg each) were used for DNA extraction using the NucleoSpin Soil kit (Macherey–Nagel, Düren, Germany). The samples were first homogenized with 0.6 to 0.8 mm ceramic beads in NucleoSpin tubes using a Taco Prep bead beater (GeneResearch Biotechnology Corp., Taichung, Taiwan). DNA extraction was performed following the manufacturer’s protocol, and the extracted DNA was stored at −20°C for future analysis. Amplification of the V4 region of the 16S rRNA gene was performed with TaKaRa Ex-Taq polymerase (TaKaRa Bio, Shiga, Japan) using universal primers (forward: 5′-GGACTACHVGGGTWTCTAAT-3′ and reverse: 5′-GTGCCAGCMGCCGCGGTAAT-3′). The PCR conditions were as follows: initial denaturation at 94°C for 3 minutes, followed by 30 cycles of 94°C for 45 seconds, 55°C for 1 minute, 72°C for 1.5 minutes, and a final extension at 72°C for 10 minutes. Amplicons were purified using a QIAquick PCR purification kit and normalized to 50 ng per sample using a Spark 10 M Multimode microplate reader (Tecan Group AG, Männedorf, Switzerland). Finally, DNA library construction and sequencing were performed on an Illumina MiSeq platform (eGenome Inc., Seoul, Korea) to generate paired-end reads of 2×300 bp.

### Microbiome analysis

Microbial community analysis was conducted using Quantitative Insights into Microbial Ecology 2 (QIIME 2) v.2021.11 (https://qiime2.org) [8], in conjunction with the SILVA 16S rRNA gene reference database [9]. To process the raw sequencing reads, individual primers and adapters were trimmed using the Cutadapt and demux plugins in QIIME 2, followed by demultiplexing with custom Perl scripts. Quality trimming, filtering, and chimeric sequence removal were performed on the demultiplexed reads using the denoise-paired option within the Divisive Amplicon Denoising Algorithm (DADA2) plugin. For DADA2, the denoise-pair settings included trimming eight base pairs from the left and truncating the reads at 180 bp. The resulting phylogenetic tree facilitated analysis of the microbial diversity of the samples. Various alpha and beta diversity metrics were derived from this phylogenetic tree using the core-metrics-phylogenetic, alpha-group-significance, and beta-group-significance functions in QIIME 2.

Alpha diversity and Faith’s phylogenetic distance were calculated to assess species richness and phylogenetic diversity within the microbiota. Beta diversity analysis was performed using non-metric multidimensional scaling (NMDS) based on the Bray-Curtis dissimilarity matrix between samples, utilizing the vegan package in R. NMDS is an ordination method used to depict data patterns in multidimensional spaces. The Adonis statistical test with 999 permutations was used to determine the effect of inherent factors on the microbial community throughout the growth stages. Unweighted UniFrac analyzes the differences in microbial composition based on the presence or absence of taxa, highlighting diversity without considering abundance. In contrast, weighted UniFrac includes both presence and relative abundance, making it sensitive to changes in the dominant taxa within the community. Amplicon sequence variants identified by DADA2 were taxonomically classified using the SILVA 132 16S rRNA classifier, employing a pre-trained classifier using QIIME 2’s fit-classifier-naïve Bayes option.

### Machine learning approach for microbiome data prediction

To predict sample metadata values using the microbiome data, we utilized the q2-sample-classifier plugin from QIIME 2 (version 2021.11). The Random Forest algorithm was selected because of its capability to manage high-dimensional data and identify complex interactions among variables. We employed 5-fold cross-validation for all models, to train, evaluate, and predict the data sampled from the dataset. This method involved splitting the dataset into five equal portions. The process was repeated five times, with four subsets used for training and the remaining subset used for evaluation each time. The evaluation subset was rotated in each iteration to ensure that all data were used for both training and evaluation. This approach involved training multiple decision trees on random subsets of data, with their aggregated predictions enhancing overall model accuracy. Feature importance scores were computed to identify the most significant predictors in the dataset.

### Metagenomics prediction

Metagenomic function was predicted using Phylogenetic Investigation of Communities by Reconstruction of Unobserved States (PICRUSt), which utilizes 16S rRNA marker gene sequences and references to previously published complete genome sequences. To normalize the data, the 16S rRNA gene copy numbers were performed rarefaction to 5,500 in the BIOM files. Subsequently, metagenomes were forecasted using Kyoto Encyclopedia of Genes and Genomes (KEGG) pathways. The predicted metagenomes were then grouped according to a particular hierarchical level within the KEGG pathway, utilizing its associated metadata. Principle component analysis (PCA) was conducted using STAMP version 2.1.3.

### Statistical analysis

Statistical analyses were performed using R. One-way ANOVA was used to compare microbial abundance between groups. Post-hoc Tukey’s honest significant difference tests were used to test for pairwise multiple comparisons. Statistical significance was set at p<0.05. To evaluate the relationship between microbial abundance and body weight, simple linear regression was performed to calculate Pearson’s correlation coefficient (R) along with the corresponding p-values. For the heatmap, the relative abundance of the 50 most abundant genera was normalized using z-scores for comparison across growth stages. Power analysis showed sufficient statistical power for feces (n = 133, >0.80) and ileal content (n = 64, 0.70 to 0.75) data with a medium effect size (Cohen’s f = 0.25) and α = 0.05.

## RESULTS

### Animal growth

**Figure 1:**
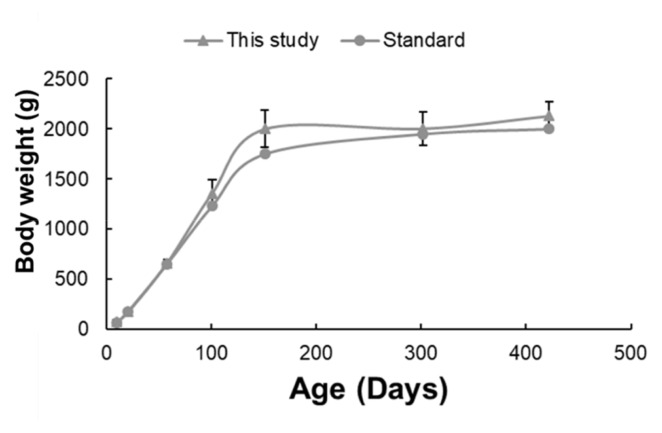
Chicken body weight at various growth stages. Body weight comparison between the standard Hy-Line Brown chickens and the chickens analyzed in this study. The standard Hy-line weight represents the typical growth in the management guide. The study presents the average values for each chicken, with standard deviations represented by error bars.

Initially, both groups showed a rapid increase in body weight, with the growth rate stabilizing after approximately 200 days. The study group (represented by blue triangles) exhibited a slightly higher body weight than the standard group (represented by orange circles) from approximately 100 d. The data for the standard group were obtained from the Hy-Line website. However, the difference narrowed, and both groups converged around the 400-day mark. The mean chicken body weights at different growth stages were 2,001.69±187.53 g on day 151, 2,005.97±168.144 g on day 302, and 2,132.44±139.54 g on day 422 (Figure 1).

### Sequencing gut microbiome

After sequencing the 16S rRNA and performing quality control of the sequences, 2,902,168 sequences (mean = 37,207± 16,277) were obtained. Each feces sample generated 36,980± 17,947 reads on average, with each sample of ileal contents generating 37,960±12,317 reads. The reads generated in feces samples of different growth stages were 36,169±22,620 on day 151, 41,257±14,901 on day 302, and 33,449±14,029 on day 422. Similarly, for the ileal contents at different growth stages, the reads generated were 45,791±16,689 on day 151, 37,230± 7,127 on day 302, and 30,107±6,527 on day 422.

### Alpha diversity of gut microbiota

**Figure 2:**
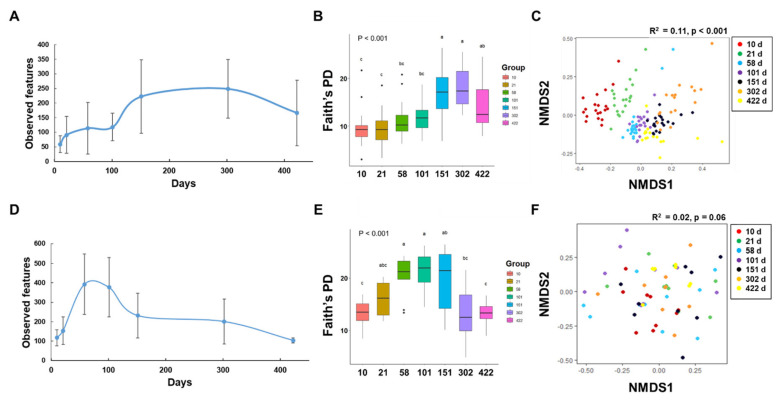
Microbial communities in feces and ileal contents were analyzed across different growth stages. Rarefaction curves representing observed features in feces (A) and ileal contents (D). Alpha diversity, measured by Faith's phylogenetic diversity, is shown for feces samples (B) and ileal contents (E). Statistical comparisons were performed using one-way ANOVA with Tukey's post-hoc test. NMDS based on Bray–Curtis dissimilarity was applied to gut microbiota across seven age groups for feces samples (C) and ileal contents (F). Statistical significance was assessed using the Adonis test with 999 permutations. ^a–c^ Superscript letters within a figure denote significant differences (p<0.001). NMDS, non-metric multidimensional scaling.

Community diversity was explored to investigate the microbiomes of developing laying hens. Microbial communities in the feces and ileal contents exhibited distinct patterns of richness and diversity over time. The richness of the feces microbiota increased until 302 d of age and then decreased (Figure 2A). In contrast, the species richness of the ileal samples increased until 58 days of age and then decreased (Figure 2D). Furthermore, the microbial diversity of the ileal samples, as measured by Faith’s phylogenetic diversity, significantly increased with the progression of the growth stages. In contrast, the ileal content increased until 151 d and then decreased (p<0.001; Figure 2E).

### Beta diversity of gut microbiota

NMDS was employed to analyze and interpret microbial community structure patterns. This technique offers insights into sample similarities and differences by leveraging non-linear relationships and distance-based metrics for visualization. The NMDS plot revealed that the feces samples could be categorized into seven discrete groups based on their microbial composition: 10, 21, 58, 101, 151, 302, and 422 d However, no cluster formation was observed in the ileal contents. Adonis statistical analysis indicated a significant association between the growth stage of chickens and the composition of their intestinal microbiota (feces: R^2^ = 0.11, p<0.001; ileal contents: R^2^ = 0.02, p = 0.06; Figures 2C, 2F). Regardless of the weighted UniFrac plot revealing the sampling location, there was a tendency to cluster, and in unweighted UniFrac, the early growth stages tended to be clustered in feces; however, in ileal contents, 422 days also tended to be clustered (Supplements 4, 5).

### Machine learning of gut microbiota by growth stages

**Figure 3:**
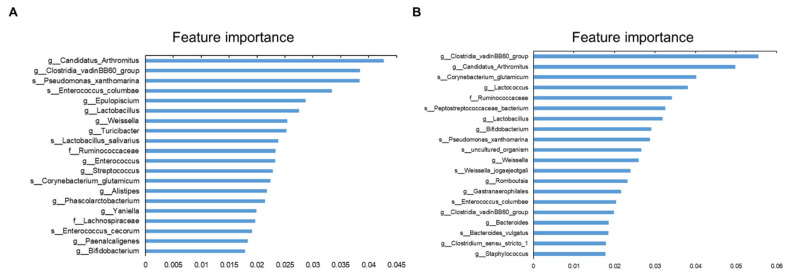
Feature importance plots highlighting the top 20 genera for predicting chicken age from feces (A) and ileal contents (B). The genus with the highest significance is ranked with an importance score in the plot.

Non-linear changes in microbial composition during chicken growth can be identified using machine learning algorithms, and chicken age can be predicted by learning in growth-specific microbial communities. A total of 133 samples and 2,477 features were trained on the feces contents. Similarly, 65 samples and 2,252 features were trained on the ileal contents. We identified the 20 most important microbiomes associated with each growth stage (Figure 3). Regardless of the sample site, *Candidatus* Arthromitus and *Clostridia vadinBB60 group* had the highest scores. The model accuracy was 0.969 for feces and 0.984 for ileal contents. Receiver Operating Characteristic curves showed classification performance with an area under the curve of 1.00, regardless of the sampling site (Supplements 6, 7).

### Assessing the composition of gut microbiota by 16S rRNA sequencing

To characterize the microbiota in detail, we performed a differential analysis to evaluate the relative abundance of phyla and genera. The leading three phyla in the feces and ileal contents were Firmicutes, Proteobacteria, and Bacteroidota (Table 2). Firmicutes showed the highest relative abundance in both groups. Unusually, Fusobacteriota showed a significantly higher relative abundance in the feces at 422 d (Table 2).

**Figure 4:**
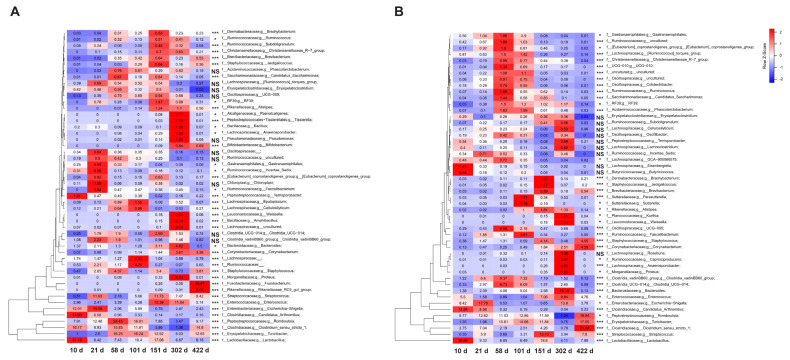
A heatmap displaying the 50 genera with the highest abundance across seven growth stages in feces (A) and ileal contents (B). Each cell represents the average relative abundance, normalized by genus, with colors indicating z-scores (red for higher abundance and blue for lower abundance). Asterisks denote levels of statistical significance: * p<0.05, ** p<0.01, *** p<0.001, and NS indicates no significance (p≥0.05).

**Figure 5:**
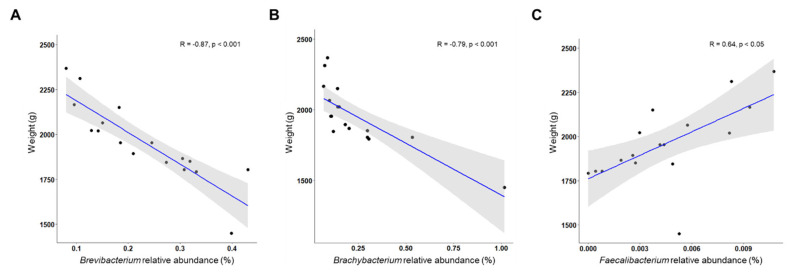
The relationship between feces relative abundance and body weight at 302 days. To assess the relationship between relative abundance and body weight, simple linear regression was employed to calculate Pearson's correlation coefficient (R) and corresponding p-values.

At the genus level, 457 genera were detected in the feces, and 415 were detected in the ileal contents. We selected the 50 genera with the highest abundance (Figure 4). In feces, *Lactobacillus, Romboutsia, Clostridium_sensu_stricto_1, Turicibater, Escherichia-Shigella, Streptococcus, Enterococcus, Candidatus* Arthromitus, *Staphylococcus*, and *Bacteroides* were the most abundant. Among them, the top five showed similar results to those of our previous study, but *Streptococcus* showed high values at 21 and 151 d, whereas *Enterococcus* increased until 151 d and then decreased (p<0.05). *Fusobacterium* within Fusobacteriota had the highest abundance at day 422 (p<0.05). In ileal contents, the top 10 genera were observed to be similar to those in feces, but *Turicibacter* and *Clostridium_sensu_stricto_1* had the highest abundance at 422 d. *Bacteroides* and *Enterococcus* were more abundant at 302 d than at the other growth stages. *Proteus* and *Anaerosporobacter* had the highest abundances at day 302 (p<0.05). At 322 days, in feces samples, *Brevibacterium* (R= −0.87; p<0.001) and *Brachybacterium* (R= −0.79; p<0.001) were negatively correlated with body weight, whereas *Facalibacterium* was positively correlated (R = 0.64; p<0.05; Figure 5).

### Functional profiling of metagenomic abundance

Metagenomic characteristics were predicted using the 16S rRNA sequence data to detect metagenomic shifts across different growth stages. A total of 6,866 KEGG pathways were identified in the feces, whereas 6,824 KEGG pathways were observed in the ileal contents.

**Figure 6:**
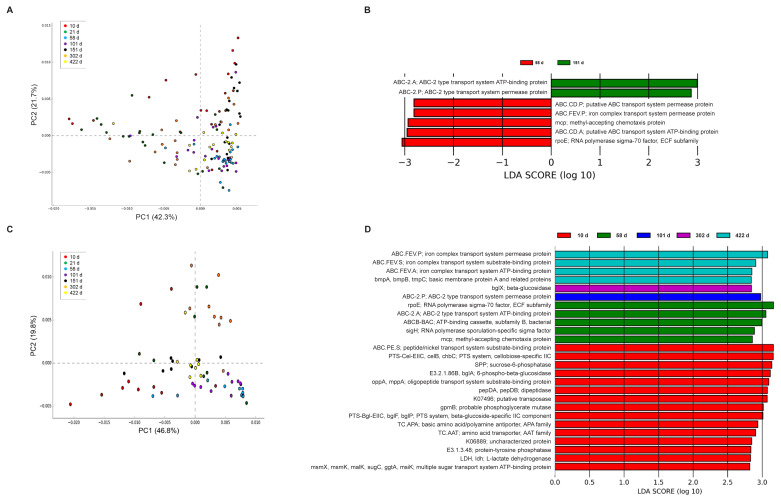
PICRUSt predicted functions at the KEGG pathway level are illustrated. The PCA plots display PICRUSt results for feces samples (A) and ileal contents (C). Linear discriminant analysis (LDA) effect size (LEfSe) analysis of gut microbiota in different growth stages for feces (B) and ileal contents (D). KEGG, Kyoto encyclopedia of genes and genomes; PCA, principle component analysis.

We conducted a Principal Component Analysis (PCA) on the KEGG pathway data to gain insights into how these pathways were distributed and varied across growth stages. The resulting PCA plot (Figures 6A, 6C) revealed that days 10, 21, and 322 were spread out, but days 58, 101, and 422 were clustered in the feces. We observed that the ileal contents tended to be clustered, except at 21 d.

We performed linear discriminant analysis effect size (LEfSe) analysis of the KEGG pathways in the feces and ileal samples to identify key functional disparities at different growth stages. In feces, linear discriminant analysis (LDA) scores were higher at 58 d than at 151 d for iron transport pathways, including “ABC.CD.P; putative ABC transport system permease protein” and “ABC.FEV.P; iron complex transport system permease protein.” The 58 d group also exhibited increased LDA scores for iron transport and chemotaxis pathways, such as “ABC.CD.P,” and stress response pathways like “rpoE; RNA polymerase sigma-70 factor, ECF subfamily.” Conversely, the 151 d group showed elevated LDA scores for ABC transport system pathways, particularly “ABC-2. A; ABC-2 type transport system ATP-binding protein,” which focuses on nutrient uptake by gut microorganisms (Figure 6B). In ileal contents, the 10 d group exhibited higher LDA scores for carbohydrate metabolism pathways, including “SPP; sucrose-6-phosphatase” and “PTS-Bgl-EIIC; beta-glucoside-specific IIC component,” compared to the late growth stages. The 58 and 101 d groups showed elevated LDA scores for ABC transport and stress response pathways in the d group. The 302 d and 422 d groups, especially the latter, exhibited the highest LDA scores for iron transport pathways such as “ABC.FEV.P” and “ABC.FEV.S”. Additionally, at day 302, bglX and beta-glucosidase showed the highest values (Figure 6D).

## DISCUSSION

Previous studies have examined the gut microbiome of laying hens up to pre-laying [7]. In the present study, we examined the gut microbiome of Hy-line brown laying hens at seven growth stages after laying. Although it is beneficial to analyze the gut microbiome before laying, the primary purpose of raising laying hens is to produce eggs; therefore, we analyzed the gut microbiome after laying. Laying hen performance and egg production are also linked to the gut microbiome. A previous study showed that the gut microbiota on the surface of eggs affects chicks shortly after hatching [10]. Similarly, changes in feed have a significant impact on the gut microbiome.

Alpha diversity is one of the most popular methods used in microbial analysis to determine the diversity of microbial communities. In general, diversity increases positively with growth and then gradually decreases; this can be observed in many animals, including humans [11,12]. Interestingly, feces increased up to 302 days of age, but ileal contents increased up to 101 days of age, then decreased. The ileal exhibits a stronger association with the immune system than the large intestine, resulting in rapid changes in the microbial composition [13]. As the immune system develops concomitantly with the growth of chickens, the microbiota typically experiences a reduction in diversity owing to competitive interactions. Therefore, microbial diversity seems to be decreasing in the ileal contents. The composition and diversity of the gut microbiota were also influenced by the fiber content in the diets. Higher fiber levels have been shown to significantly alter the gut microbiota, promoting changes in both diversity and composition [14]. In our previous study, feces tended to cluster according to feed content, and even after feeding the laying diet, they tended to cluster according to feed content. However, the ileal contents did not cluster, as the microbial species tended to be similar as they grew [15]. The high feature importance of *Clostridia_vadinBB60_group* and Candidatus_*Arthromitus* in machine learning suggests that they play an important role in predicting chicken growth stages. The relative abundance of *Clostridia_vadinBB60_group* in feces did not significantly change with growth stage, but was significantly higher in the ileal contents before laying (p<0.001). The Clostridia_vadinBB60_group is associated with the production of short chain fatty acids, and well-textured mash diets have a higher crude fiber content than starter diets [16]. In addition, Candidatus_*Arthromitus* was significantly more abundant during early growth, regardless of the sampling site (p<0.001). Candidatus_*Arthromitus* regulates intestinal immunity and prevents pathogen colonization in chickens [17]. It has also been investigated as a potential biomarker for predicting human colorectal polyps and cancer [18]. The high model accuracy suggests that age can be predicted well based on the microbiome; however, owing to the small number of ileal samples, more training data are needed in future studies. It is also necessary to analyze the pan-genome of this microbial group. Other researchers have employed machine learning algorithms such as random forests, support vector machines, and neural networks to effectively predict disease conditions, identify biomarkers, and explore the influence of diet, medications, and environmental factors on the microbiome [19]. These approaches have enhanced scientific understanding of the complex interactions between microbial communities and their hosts. Machine learning complemented the microbiological analysis by identifying key microbial predictors, such as Candidatus *Arthromitus* and Clostridia vadinBB60 group, and evaluating their consistency across sampling sites, offering a framework to understand gut microbiota dynamics and their link to growth stages.

The phylum Fusobacteriota exhibited significant representation in feces at 422 d, which is corroborated by the high prevalence of *Fusobacterium* at the genus level. Within the *Fusobacterium* genus, certain species are associated with pathogenicity in chickens [20], whereas others are implicated in lipid metabolism [21]. Senior dogs over seven years of age showed a higher abundance of *Fusobacterium* than junior dogs, indicating its potential as an aging biomarker [22]. In a previous study, *Lactobacillus* was high early in growth and then decreased, whereas *Bifidobacterium* was not observed but increased significantly after laying, which may be due to the higher calcium content of the layer feed compared to other diets. Similar results have been observed in rats [23]. Additionally, the layer diet in this study, provided in mash form, has been associated with an increase in lactic acid bacteria at 151 d, suggesting a potential link to the growth of *Lactobacillus* populations [24]. *Brevibacterium* and *Brachybacterium* are recognized to be present in poultry litter [25] and have demonstrated an increase in abundance with elevated phosphorus intake, indicating their potential utility as biomarkers [26]. Excessive phosphorus intake may adversely affect body weight by inducing a calcium-phosphorus imbalance and diminishing metabolic efficiency. However, additional research is necessary to elucidate the specific mechanisms involved [27].

The LEfSe analysis indicated that the pathways related to membrane transport, carbohydrate metabolism, and energy metabolism received the highest scores. At 151 days after switching from feces to the laying hen diet, ABC-2 type transport system related proteins had a high LDA score. These proteins play important roles in enabling the transport of minerals and metal ions across cell membranes and regulating substrate movement using adenosine triphosphate. This is thought to be influenced by the higher content of crude ash and calcium in the layer diet compared to the previous diet. Similar results to previous studies were observed at 10, 58, and 101 d. Beta-glucosidase, with a high LDA score at day 322, plays an important role in the final step of the degradation of disaccharides, such as cellulose, into glucose. In terms of relative abundance at the same age, *Cellulosilyticum* was observed to be significantly higher, which is known to degrade cellulose [28]. It has also been suggested that it plays a role in cellulose digestion and absorption in the ileal; however, no correlation with body weight was observed (R = 0.022; p = 0.96). Similarly, in the feces at day 422, the change in feed is thought to have affected the transport of proteins.

## CONCLUSION

Previous studies have focused on the gut microbiota in hens before laying, leaving a gap in our understanding of the changes that occur after egg-laying. By examining the gut microbiota from birth to approximately one year post-laying, we gathered comprehensive data on Hy-Line Brown chickens, indirectly confirming that a calcium-rich layer diet affects the gut microbiota. The machine learning model developed to predict chicken age using these data showed promise. However, the small sample size suggests that a more robust model could be developed with additional growth period data or a larger sample size in future studies. Establishing a structured framework for gut microbiota alterations during the feed transition period in layers could provide valuable insights for subsequent research.

## Figures and Tables

**Table 1:** Composition of experimental diets

Feed type	Starter	Well-textured mash	Layer
Crude protein (min) (%)	18	15	14
Crude fat (min) (%)	3	2	2
Crude fiber (max) (%)	5.5	6.5	7
Crude ash (max) (%)	8	9	17
Calcium (min) (%)	0.8	0.7	2.8
Phosphorus (max) (%)	0.9	0.9	0.9
Methionine+cystine+MHA (min) (%)	0.8	0.55	0.35
Metabolizable energy (min) (Mcal/kg)	2.92	2.65	2.4

**Table 2:** Relative abundance of phyla in feces and ileal contents^1)^

Taxon	Relative abundance (%)							p-value

	10 d	21 d	58 d	101 d	151 d	302 d	422 d	
Feces								
Firmicutes	84.6±18.2^2)^^bc^	74.3±14.5^ab^	91.4±5.33^c^	87.9±6.99^c^	87.7±5.73^c^	70.9±16.4^a^	68.8±20.5^a^	**
Proteobacteria	12.5±18.7^ac^	20.9±14.9^c^	4.13±3.89^ab^	7.91±6.78^ab^	1.94±1.48^a^	14.8±16.6^bc^	3.81±7.64^ab^	**
Bacteroidota	1.52±3.25^a^	2.21±4.08^a^	1.41±2.01^a^	1.55±1.74^a^	5.28±5.75^ab^	7.90±7.31^ab^	9.77±18.2^b^	*
Actinobacteriota	0.127±0.156^a^	0.642±0.498^ab^	1.72±1.39^ab^	2.02±1.33^bc^	3.61±1.98^cd^	5.54±3.51^d^	5.45±3.15^d^	**
Fusobacteriota	0.000±0.000^a^	0.000±0.000^a^	0.000±0.000^a^	0.000±0.000^a^	0.254±0.560^a^	0.050±0.0703^a^	11.0±16.0^b^	**
Cyanobacteria	0.358±0.552^a^	1.74±3.19^b^	0.388±0.602^a^	0.264±0.393^a^	0.421±1.04^a^	0.100±0.0975^a^	0.0705±0.0921^a^	*
Ileal contents								
Firmicutes	85.2±12.4^b^	74.6±17.0^b^	88.8±4.68^b^	85.0±5.99^b^	85.0±9.96^b^	57.6±11.9^a^	86.9±10.3^b^	**
Proteobacteria	9.16±13.8^ac^	18.8±19.5^bc^	2.02±0.891^a^	5.19±2.53^ab^	2.07±1.49^a^	21.8±13.4^c^	3.92±9.91^ab^	**
Bacteroidota	1.45±2.04^a^	2.53±2.63^a^	4.21±2.53^a^	6.68±4.05^a^	6.14±9.03^a^	16.9±10.1^b^	0.701±0.404^a^	**
Actinobacteriota	0.239±0.182^a^	0.626±0.547^a^	0.545±0.464^a^	0.846±0.677^a^	4.99±3.24^bc^	3.30±2.37^b^	6.33±2.39^c^	**
Cyanobacteria	0.726±1.21^ab^	1.13±1.24^ab^	1.92±1.81^b^	0.956±0.640^ab^	0.224±0.105^a^	0.139±0.226^a^	0.0543±0.0581^a^	*
Patescibacteria	0.0593±0.0508^a^	0.0560±0.0316^a^	1.44±1.46^b^	0.556±0.451^ab^	0.841±0.812^ab^	0.100±0.113^a^	0.0508±0.0503^a^	**

1) One-way analysis of variance with Tukey’s post-hoc test was used.

2) Data is shown as the mean±standard deviations.

a–d Within a row, different superscript letters indicate significant differences (p<0.05).

* p<0.01,

** p<0.001.

## SUPPLEMENTARY MATERIAL

**Supplement 1.** Monthly average temperature measured inside the chicken farm (ESP-01).

**Supplement 2.** Monthly egg production of laying hens.

**Supplement 3.** Sampling data for each growth stage

**Supplement 4.** Principal coordinate analysis of the microbiota across seven growth stages in feces.

**Supplement 5.** Principal coordinate analysis of the microbiota across seven growth stages in ileal contents. Weighted (A) and unweighted (B) based on UniFrac distances. PERMANOVA anaysis with 999 permutations is shown.

**Supplement 6.** The confusion matrix illustrates the model's classification performance across seven growth stages in feces, with proportions represented by color intensity (A). Receiver Operating Characteristic (ROC) curves are a graphical representation of the classification accuracy of a machine-learning model (B).

**Supplement 7.** The confusion matrix illustrates the model's classification performance across seven growth stages in ileal contents, with proportions represented by color intensity (A). Receiver Operating Characteristic (ROC) curves are a graphical representation of the classification accuracy of a machine-learning model (B).
