# Supplementary figures and images for: A longitudinal investigation of gut microbiota dynamics in laying hens from birth to egg-laying stages
Source: Anim Biosci. 2025 Apr 11;38(8):1773–83. doi: 10.5713/ab.24.0889 (PMC12229937; doi:10.5713/ab.24.0889)

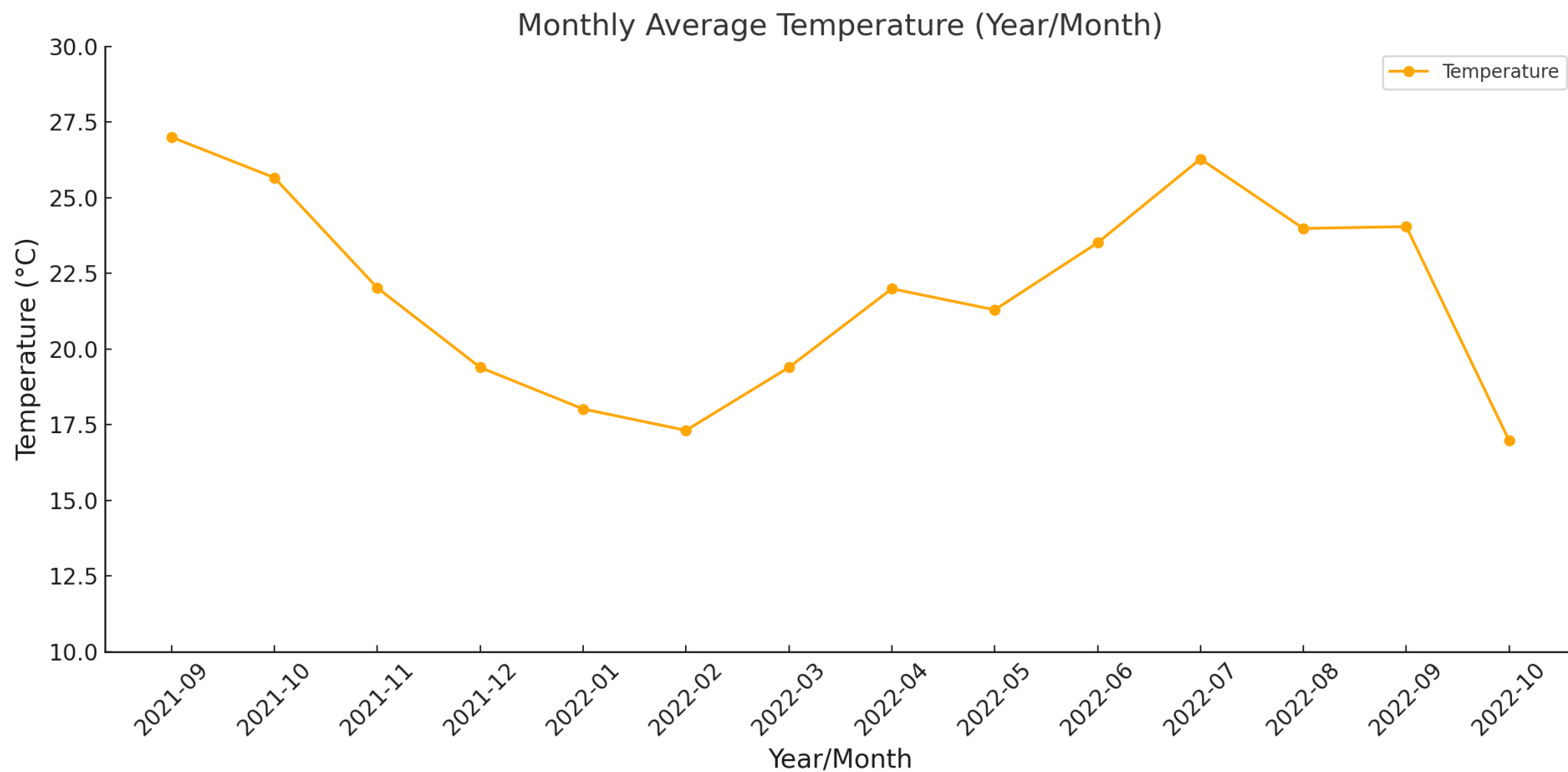

**Supplement 1.** Monthly average temperature measured inside the chicken farm (ESP-01).

Supplement: Supplementary file 1 [file ab-24-0889-Supplementary-1.pdf]
