# Supplementary material for: A longitudinal investigation of gut microbiota dynamics in laying hens from birth to egg-laying stages
Source: Anim Biosci. 2025 Apr 11;38(8):1773–83. doi: 10.5713/ab.24.0889 (PMC12229937; doi:10.5713/ab.24.0889)
Supplement: Supplementary file 2 [file ab-24-0889-Supplementary-2.pdf]

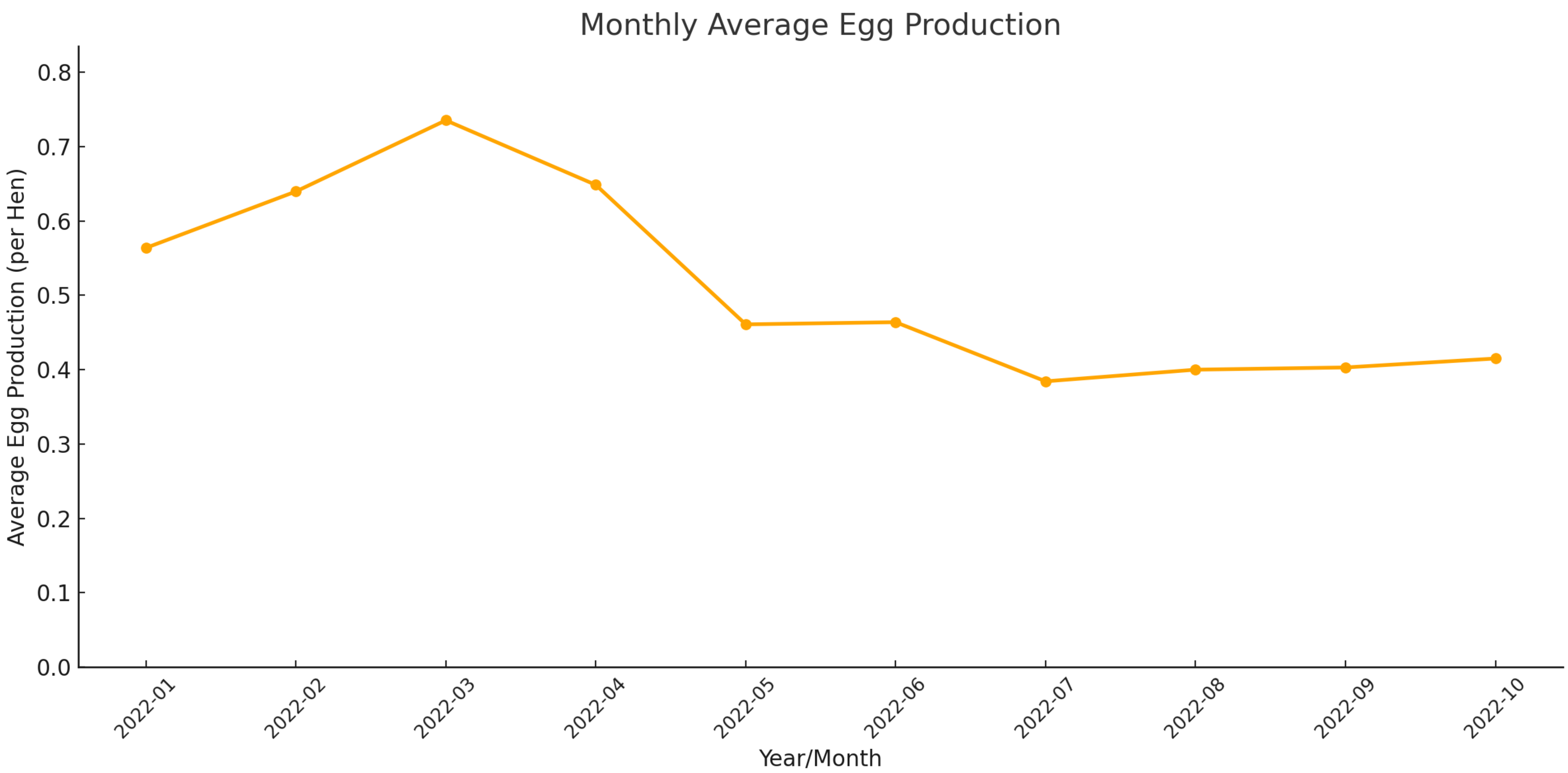

Supplement 2. Monthly Egg Production of Laying Hens. The total number of eggs collected was divided by the number of hens to calculate the average egg production per hen.
