# Supplementary material for: A longitudinal investigation of gut microbiota dynamics in laying hens from birth to egg-laying stages
Source: Anim Biosci. 2025 Apr 11;38(8):1773–83. doi: 10.5713/ab.24.0889 (PMC12229937; doi:10.5713/ab.24.0889)
Supplement: Supplementary file 3 [file ab-24-0889-Supplementary-3.pdf]

**Supplement 3**. Sampling data for each growth stage.

| Age | Sampling dates | Number of feces | Number of ileal contents | Feed |
|---|---|---|---|---|
| 10 | 09/2021 | 20 | 10 | Starter |
| 21 | 09/2021 | 20 | 9 | Starter |
| 58 | 10/2021 | 20 | 10 | Well-textured mash |
| 101 | 12/2021 | 20 | 10 | Well-textured mash |
| 151 | 01/2022 | 20 | 9 | Layer |
| 302 | 06/2022 | 17 | 9 | Layer |
| 422 | 10/2022 | 16 | 9 | Layer |
