# Supplementary material for: A longitudinal investigation of gut microbiota dynamics in laying hens from birth to egg-laying stages
Source: Anim Biosci. 2025 Apr 11;38(8):1773–83. doi: 10.5713/ab.24.0889 (PMC12229937; doi:10.5713/ab.24.0889)
Supplement: Supplementary file 4 [file ab-24-0889-Supplementary-4.pdf]

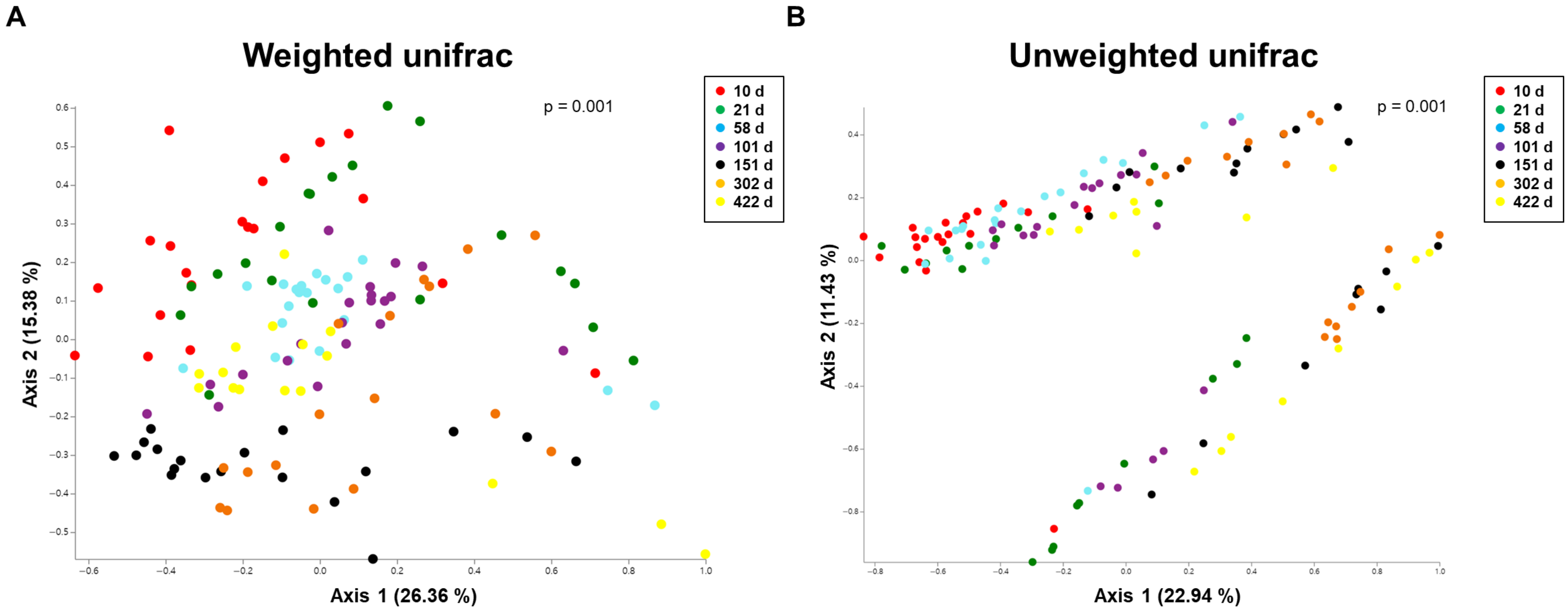

Supplement 4. Principal coordinate analysis of the microbiota across seven growth stages in feces. Weighted (A) and unweighted (B) based on UniFrac distances. PERMANOVA anaysis with 999 permutations is shown.
