# Supplementary material for: A longitudinal investigation of gut microbiota dynamics in laying hens from birth to egg-laying stages
Source: Anim Biosci. 2025 Apr 11;38(8):1773–83. doi: 10.5713/ab.24.0889 (PMC12229937; doi:10.5713/ab.24.0889)
Supplement: Supplementary file 5 [file ab-24-0889-Supplementary-5.pdf]

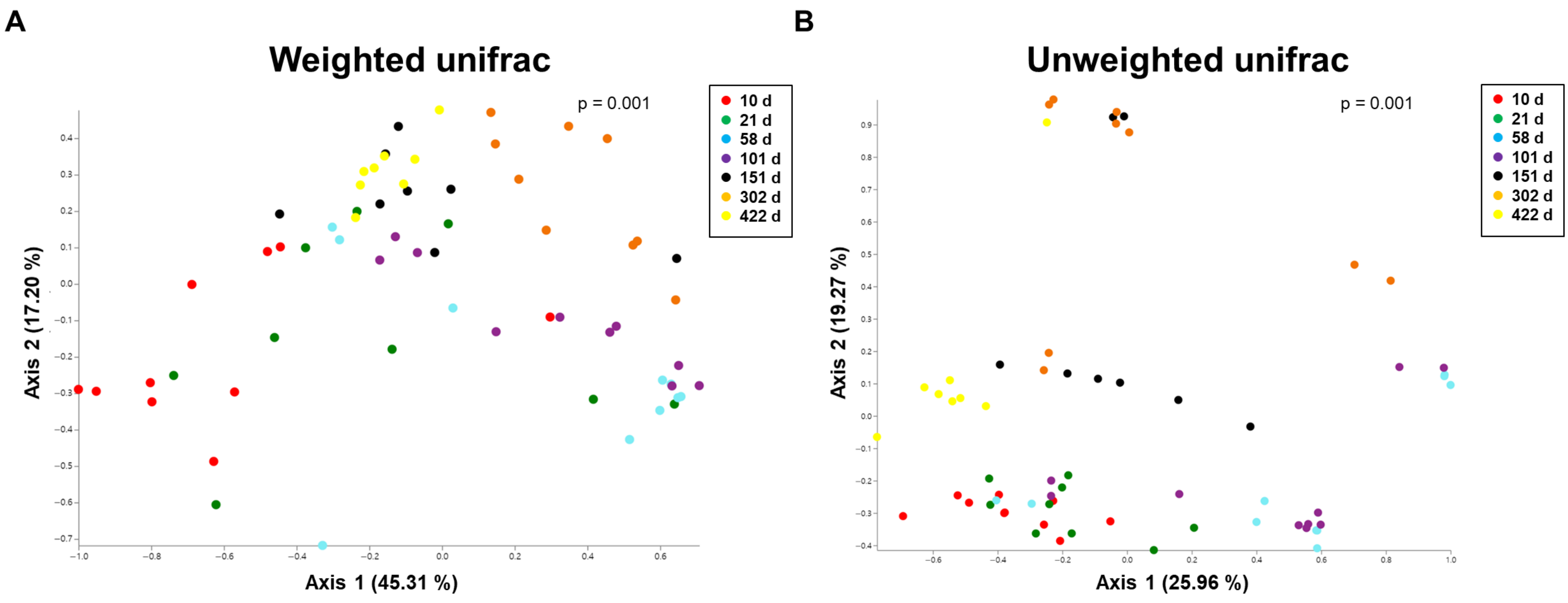

Supplement 5. Principal coordinate analysis of the microbiota across seven growth stages in ileal contents. Weighted (A) and unweighted (B) based on UniFrac distances. PERMANOVA anaysis with 999 permutations is shown.
