# Supplementary material for: A longitudinal investigation of gut microbiota dynamics in laying hens from birth to egg-laying stages
Source: Anim Biosci. 2025 Apr 11;38(8):1773–83. doi: 10.5713/ab.24.0889 (PMC12229937; doi:10.5713/ab.24.0889)
Supplement: Supplementary file 6 [file ab-24-0889-Supplementary-6.pdf]

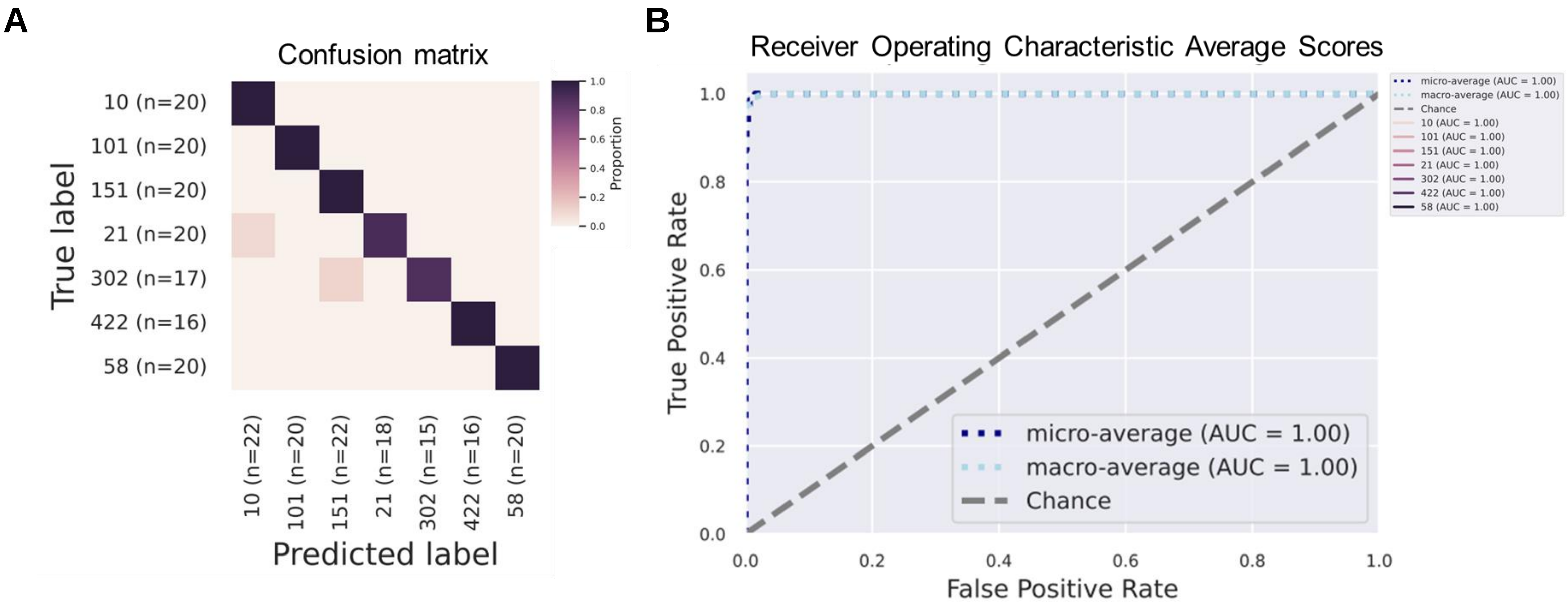

Supplement 6. The confusion matrix illustrates the model's classification performance across seven growth stages in feces, with proportions represented by color intensity (A). Receiver Operating Characteristic (ROC) curves are a graphical representation of the classification accuracy of a machine-learning model (B).
